# Stereotactic Radiosurgery for Patients With Five or More Brain Metastases: Retrospective Single-Institution Analysis of Prognostic Factors

**DOI:** 10.7759/cureus.99268

**Published:** 2025-12-15

**Authors:** Michael T Milano, Dandan Zheng, Jihyung Yoon, Yuwei Zhou, Hyunuk Jung, Haoming Qiu, Terris Igwe, Daniel Huang, Sara Hardy, Kenneth Usuki

**Affiliations:** 1 Radiation Oncology, University of Rochester, Rochester, USA; 2 Radiation Oncology, University of Washington, Seattle, USA

**Keywords:** brain metastases, brain metastasis velocity, grade prognostic assessment, radiosurgery, treatment of multiple brain metastases

## Abstract

Purpose: We describe patient outcomes and prognostic factors following linear accelerator-based stereotactic radiosurgery (SRS) for five or more brain metastases, without prior or planned whole-brain radiotherapy.

Methods: We identified 116 eligible patients treated with SRS from 2019 to 2024 for newly diagnosed brain metastases. We describe clinical factors associated with survival ≤2 months and analyze brain metastasis velocity measured as the number (BMV) or volume (vBMV) of new metastases per year.

Results: The number of treated brain metastases ranged from 5 to 41 (median 10); net lesion volume ranged from 0.1 to 59.8 (median 5.2) cc. Primary cancers included non-small cell lung (n=65), melanoma (n=20), breast (n=19), kidney (n=6), gastrointestinal (n=4), and other (n=4) cancers. The 6-, 12-, and 24-month overall survival (OS) was 60.3%, 40.5%, and 28.0%, respectively. A progressive extracranial disease at the time of brain metastases and lower predicted survival from graded prognostic assessments (GPAs) were significantly adverse factors for OS on multivariable Cox regression and were associated with ‘poor survivors’ who died ≤2 months from SRS (n=21) or at >2 months but opting against post-SRS cancer care and follow-up imaging (n=6; OS=2.1-5.8 months). Forty-two patients developed new brain metastases after SRS, while 28 (after ≥6-month follow-up) did not. Among these patients, OS was significantly associated with BMV and vBMV, though vBMV was not significant on multivariable Cox regressions that included BMV.

Conclusions: For patients with five or more brain metastases, clinical factors, including the status of extracranial disease and GPA, can potentially aid in selecting patients best-suited for SRS for multiple brain metastases, versus potentially deferring SRS in favor of supportive care. While vBMV is associated with OS, BMV appears more prognostic.

## Introduction

Stereotactic radiosurgery (SRS) is an accepted standard of care for multiple brain metastases, though appropriate selection of patients better-suited for SRS over whole-brain radiotherapy (WBRT), close observation with serial imaging, or supportive care alone are not well-characterized [1]. Because of adverse effects on neurocognitive function and quality of life (QOL) [1], delays in systemic therapy, and acute risks (alopecia, fatigue, otitis media, scalp irritation) with WBRT, SRS is often preferred over WBRT. Randomized controlled studies [2-6], along with meta-analyses and systematic reviews [7-9], have demonstrated no detriment in overall survival (OS) and better neurocognitive function and QOL when omitting WBRT in patients with one to four brain metastases treated with SRS (with two studies also allowing resection). MD Anderson Cancer Center reported similar findings from a randomized study of SRS vs WBRT among 72 patients with four to 15 brain metastases from non-melanoma cancers [10]. A multicenter, phase 3 randomized trial of 196 patients with five to 20 (median 14) brain metastases reported fewer symptoms, less functional decline, and similar OS with SRS vs hippocampal-avoidant WBRT [11]. Many other studies, including pooled analyses with thousands of patients [12-14], have examined SRS without WBRT for patients with many brain metastases; these data continue to evolve [1,15-17]. The number and (seemingly more so) volume of brain metastases impact outcomes after SRS [1,18], though how these factors impact treatment decision-making remains unclear, particularly in the context of other potentially prognostic factors.

As risks of new brain metastases are greater after SRS vs WBRT, salvage therapy (often SRS) is frequently required after SRS [1]. Researchers from Wake Forest University described brain metastasis velocity (BMV), the cumulative number of new brain metastases over time, as a predictor of OS, neurologic death, and receipt of salvage WBRT; lower BMV, binned into <4, 4-13, and >13 lesions/year subgroups, was associated with significantly better OS [19]. A pooled analysis of 2,829 patients from the United States (US) validated these results [20]. Yamamoto and colleagues from Japan evaluated 833 patients treated with SRS, over two to four courses, and showed BMV as prognostic following each SRS course [21]. In the US studies, most patients initially had one brain metastasis (~90% initially had four or fewer) [19,20], while the Japanese study [21] did not specify the number of lesions treated during the first SRS course.

Those who develop new brain metastases amenable to salvage therapies survive long enough to be candidates for additional treatment. At the opposite end of the spectrum are patients who die shortly after SRS. The main objectives of this study are to examine causes of death and prognostic factors associated with survival (and early death) in patients treated with SRS for five or more brain metastases. We hypothesized that median OS predicted from grade prognostic assessments (GPAs) [22-27] and extracranial progression at the time of initial brain metastases diagnosis would be prognostic for OS as well as prognostic factors for early death. Additionally, we sought to examine BMV in patients presenting with five or more lesions who survive long enough to develop new brain metastases and hypothesized that volume-based brain metastases velocity measures (vBMV) [28] would also be prognostic.

This article was previously posted to the ResearchSquare preprint server on October 21, 2025.

## Materials and methods

Patients

Patients who underwent SRS (with no prior WBRT) for five or more brain metastases, during their first or only course of SRS for all brain metastases, were retrospectively identified from a prospective database of patients treated at the University of Rochester from November 2018 through October 2024. We included patients who had undergone brain metastasis resection just prior to SRS and excluded patients with small-cell lung cancer (SCLC). We followed patients through September 2025, for ≥1 year or until death. This study was approved by the University of Rochester Research Subjects Review Board (RSRB), which serves as their Institutional Review Board (IRB).

Treatment

Treatment with SRS is described in more detail in prior publications [17,29,30] and briefly summarized here. Patients were immobilized with a BrainLAB® mask. Planning computed tomography (CT) and magnetic resonance imaging (MRI) images were imported into BrainLAB’s Multi-Metastases Elements (MME)® platform (Brainlab, Munich, Germany) and deformably registered. Gross tumor volumes (GTVs) were expanded 1-2 mm to create planning target volumes (PTVs). Treatment was planned and delivered in one, three, or five fractions (at the treating physicians’ discretion) with dynamic conformal arcs using BrainLAB MME® planning software or with volume-modulated arc therapy using Varian Eclipse® planning software, depending on target coverage and brain exposures [30]. We aimed for >95% of PTVs receiving the prescribed dose (with >94% considered acceptable). Organ-at-risk considerations were described previously [17]. Treatment was delivered on a Varian Edge® linear accelerator (Varian Medical Systems, Palo Alto, USA) equipped with BrainLAB ExacTrac® oblique orthogonal x-ray imagers and a six-degree-of-freedom robotic couch.

Data analysis

We retrospectively reviewed and described characteristics of patients, their cancer, and treatment course (including salvage therapies), along with intracranial control and OS after SRS. T-tests and chi-square tests were used to assess potential differences in variables. BMV and vBMV were calculated as the number and volume (net GTV), respectively, of new metastases, divided by the duration of time since completion of SRS. For those with innumerable (50+) new lesions, number and volume of brain metastases were estimated based on tallying all of the lesions that were seen on MR imaging (recognizing that with such large numbers, some will have been missed) and estimating volumes based on the typical size of these lesions.

OS, measured from the date of SRS to the date of death or last follow-up, was calculated by Kaplan-Meier methods. Log-rank tests and Cox regression were used to assess potential factors associated with OS. Serial MRI and clinical follow-up typically occurred every 2-3 months after SRS. Local control was measured from the date of SRS completion to the date of local recurrence or last brain imaging. Determining local recurrence (vs post-treatment changes) was based on growth on serial imaging, MR perfusion (and occasionally spectroscopy), and consensus opinion in a dedicated brain metastasis tumor board. The cause of death was determined based on review of imaging and clinical records.

## Results

We identified 164 patients who completed SRS for ≥5 brain metastases during the study period. Patients were excluded for: prior SRS for brain metastases (n=29); prior WBRT (n=6); or SCLC diagnosis (n=13). Of the remaining 116 patients, with 1,457 brain metastases, primary diagnoses included non-small cell lung (NSCLC; n=65), melanoma (n=20), breast (n=19), kidney (n=6), gastrointestinal (n=4), and other (n=4) cancers. Seven underwent resection of a brain metastasis shortly before SRS. The number of brain metastases targets ranged from five to 41 (median 10).

Survival

Table 1 outlines patient, cancer, and treatment (including the prescribed dose and fractionation) characteristics. Follow-up after SRS ranged from 0.4 to 74.7 (median 8.2) months and 12.1 to 74.7 (median 39.2) months for 22 patients alive at last follow-up. Among those with NSCLC vs all others, 41 (65%) vs. 15 (28%) had, at the time of brain metastases diagnosis, either newly diagnosed cancer or newly diagnosed extracranial metastases. There was no discernible relationship between the volume and number of brain metastases, with linear regression R2≈0. GPA-predicted median OS increased with increasing Karnofsky performance status (KPS), as expected (since GPAs incorporate KPS), albeit with linear regression R2≈0.23.

**Table 1:** Patient, cancer and treatment characteristics. SRS: stereotactic radiosurgery; KPS: Karnofsky Performance Scale; GPA: graded prognostic assessment; CNS: central nervous system; GTV: gross target volume ¥ Net GTV for the initial SRS course * includes duodenum (n=1), rectum (n=1) and esophagus (n=2) for which the gastrointestinal cancer GPA was used; also includes endometrial (n=2), ovarian (n=1) and head and neck (n=1) cancers for which the non-disease site specific GPA was used § major and minor neurologic deficits imply neurologic deficits (including headache but not seizure) requiring vs not requiring (respectively) hospitalization. All those with major deficits received dexamethasone ≥4 mg daily, as did all but one who had had seizure. ‡ 2 patients with no evidence of disease † some patients received drug prior to and after SRS resulting in net totals >100% for these cells

	All patients	Patients with no imaging follow-up and/or survival ≤2 months	Patients with imaging follow-up and survival >2 months	t-test value	chi-square value	p-value
Number of patients	116	27	89			
Age						
Range (median)	33.5-95.2 (66.6) years	41.2-83.5 (68.3) years	33.5-95.2 (65.6) years	1.256		0.21
KPS						
Range (median)	40-100 (80)	50-90 (80)	40-100 (80)	-3.342		0.001
40-60	20 (17.2%)	10 (37.0%)	10 (11.2%)		9.665	0.002
70-80	51 (44.0%)	13 (48.1%)	38 (42.7%)		0.250	0.62
90-100	45 (38.8%)	4 (14.8%)	41 (46.1%)		8.521	0.004
Neurologic symptoms						
None	52 (44.8%)	11 (40.7%)	41 (46.1%)		0.238	0.63
Minor deficits §	37 (31.9%)	11 (40.7%)	26 (29.2%)		1.267	0.26
Dexamethasone ≥4mg daily	25	8	17			
Major deficits §	17 (14.7%)	3 (11.1%)	14 (15.7%)		0.353	0.55
Seizure	7 (6.0%)	1 (3.7%)	6 (6.7%)		0.337	0.56
Major deficits OR dexamethasone ≥4mg daily	47 (40.5%)	12 (44.4%)	35 (39.3%)		0.225	0.64
Cancer type and histology						
Non-small cell lung cancer	63 (54.3%)	11 (40.7%)	52 (58.4%)		2.611	0.11
Cutaneous melanoma	20 (17.2%)	5 (18.5%)	15 (16.9%)		0.040	0.84
Breast adenocarcinoma	19 (16.4%)	3 (11.1%)	16 (18.0%)		0.713	0.40
Kidney – renal cell carcinoma	6 (5.2%)	3 (11.1%)	3 (3.3%)		2.530	0.11
Other *	8 (6.9%)	5 (18.5%)	3 (3.3%)		6.659	0.010
Status of extracranial disease						
No evidence of disease or no progression	19 (16.4%) ‡	2 (7.4%)	17 (19.%) ‡		2.068	0.15
Progression	41 (35.3%)	15 (55.6%)	26 (29.2%)		6.290	0.012
New metastatic cancer diagnosis	56 (48.3%)	10 (37.0%)	46 (51.7%)		1.780	0.18
Predicted median survival from GPAs						
Range (median)	2.0-30.0 (6.0) months	2.6-15.0 (6.0) months	2.0-30.0 (8.3) months	-2.771		0.007
≤6.0 months	63 (54.3%)	20 (74.1%)	43 (48.3%)		5.540	0.019
>6-12 months	10 (8.6%)	2 (7.4%)	8 (9.0%)		0.066	0.8
>12 months	43 (37.0%)	5 (18.5%)	38 (42.7%)		5.191	0.023
Small molecule inhibitor †						
None	72 (62.0%)	17 (63.0%)	55 (61.8%)		0.012	0.91
Before SRS	20 (17.2%)	7 (25.9%)	13 (14.6%)		1.860	0.17
After SRS	33 (28.4%)	3 (11.1%)	30 (33.7%)		5.197	0.023
Immune checkpoint inhibitor †						
None	42 (36.2%)	17 (63.0%)	25 (28.1%)		10.907	<0.001
Before SRS	44 (37.9%)	9 (33.3%)	35 (39.3%)		0.316	0.57
After SRS	57 (49.1%)	4 (14.8%)	53 (60.0%)		16.588	<0.001
CNS penetrant antibody-drug conjugate or chemotherapy †						
None	99 (85.3%)	26 (96.3%)	73 (82.0%)		3.374	0.066
Before SRS	12 (10.3%)	1 (3.7%)	11 (12.3%)		1.673	0.20
After SRS	15 (12.9%)	1 (3.7%)	14 (15.7%)		2.661	0.10
SRS – dose and fractionation						
6-7 Gy x 5	9 (7.7%)	0	9 (10.1%)		2.960	0.085
7-9 Gy x 3 (mostly 9 Gy x 3)	100 (86.2%)	26 (96.3%)	74 (83.1%)		3.013	0.083
20 Gy x 1	7 (6.0%)	1 (3.7%)	6 (6.7%)		0.337	0.56
Number of metastases						
Range (median)	5-41 (10)	5-37 (10)	5-41 (9)	1.017		0.31
5-10	68 (58.6%)	15 (55.6%)	53 (59.6%)		0.136	0.71
11-20	30 (25.9%)	6 (22.2%)	24 (26.9%)		0.243	0.62
20-30	11 (9.5%)	4 (14.8%)	7 (7.9%)		1.166	0.28
>30	7 (6.0%)	2 (7.4%)	5 (5.6%)		0.117	0.73
Net GTV ¥ of metastases						
Range (median)	0.1-59.8 (5.2) cc	0.4-53.3 (5.5) cc	0.1-59.8 (5.0) cc	0.567		0.57
≤2 cc	28 (24.1%)	5 (18.5%)	23 (25.8%)		0.607	0.44
>2-5 cc	29 (25.0%)	7 (25.9%)	22 (24.7%)		0.016	0.9
>5-10 cc	26 (22.4%)	7 (25.9%)	19 (21.3%)		0.250	0.62
>10-20 cc	22 (19.0%)	4 (14.8%)	18 (20.2%)		0.395	0.53
>20 cc	11 (9.5%)	4 (14.8%)	7 (7.9%)		1.166	0.28

**Figure 1:**
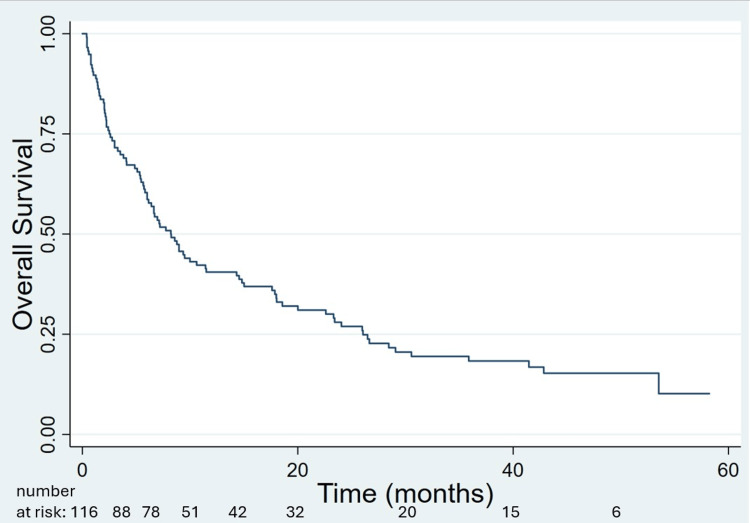
Kaplan-Meier overall survival of the entire cohort of 116 patients treated with stereotactic radiosurgery (SRS) alone for five or more brain metastases.

**Figure 2:**
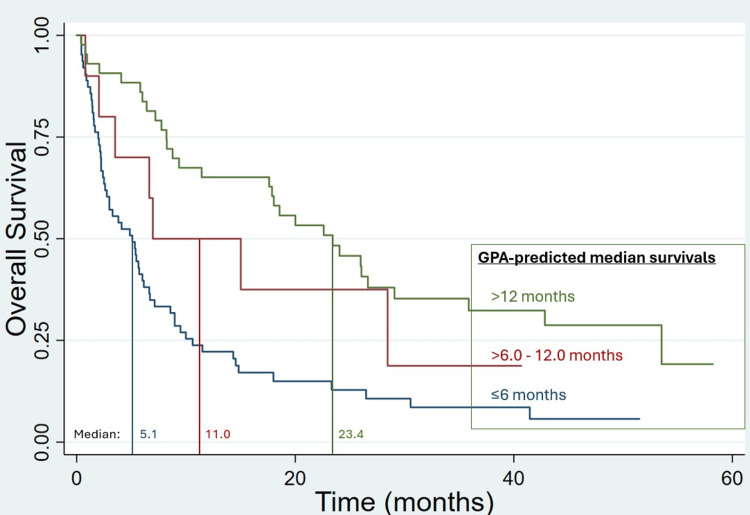
Kaplan-Meier overall survival after stereotactic radiosurgery (SRS) alone for five or more brain metastases, grouped by predicted survivals from graded prognostic assessments (GPAs).

Median OS was 8.2 months; 6-, 12- and 24-month OS was 60.3%, 40.5%, 28.0%, respectively (Figure 1). GPA-predicted median OS was significantly associated with OS as continuous (HR=0.905 per month; p<0.0001) and discrete variables (p<0.00001; Figure 2); KPS and status of extracranial disease at time of brain metastasis diagnosis were also significant factors, whereas number and volume of brain metastases were not (Table 2). While those with net GTV>20 cc fared poorly, this subgroup was small (n=11). Progressive extracranial disease at the time of brain metastases diagnosis and GPA-predicted median OS remained significant on multivariable Cox regression (Table 2). Among 63 patients with GPA-predicted median OS ≤6 months, those without (n=38) vs with (n=25) progressive extracranial disease at time of brain metastases diagnosis had median OS of 5.7 vs 3.3 months (p=0.048), respectively.

**Table 2:** Patient, cancer and treatment factors evaluated for potential effect on overall survival. KPS: Karnofsky Performance Scale; GPA: graded prognostic assessment; GTV: gross target volume; HR [95% CI]: hazard ratio with 95% confidence intervals; NED: no evidence of disease (n=2); OS: overall survival ¥ Net GTV for the initial SRS course * analysis not done (comparison focused on progressive extracranial disease vs no progression at the time of brain metastases)

	Median OS	Univariable	Univariable Cox regression	Multivariable Cox regression (model 1)	Multivariable Cox regression (model 2)
	(months)	log-rank p value	HR [95% CI] (p value)	HR [95% CI] (p value)	HR [95% CI] (p value)
Age		0.36	1.015 [0.996-1.035] (p=0.12)	Not included in the model	Not included in the model
≤60 years	11.5				
>60-70 years	7.1				
>70 years	6.1				
KPS: range (median)		0.0001	0.971 [0.957-0.984] (p<0.0001)	0.983 [0.967-0.999] (p=0.040)	Not included in the model
40-60	2.2				
70-80	6				
90-100	18.6				
Neurologic symptoms			Not applicable	Not included in the model	Not included in the model
None	7.8	0.36			
Minor deficits (see Table 1)	7	0.21			
Major deficits (see Table 1)	9	0.56			
Major deficits OR dexamethasone ≥4mg daily	7	0.66			
Cancer type and histology			Not applicable	Not included in the model	Not included in the model
Non-small cell lung cancer	8.6	0.85			
Cutaneous melanoma	12.1	0.36			
Breast adenocarcinoma	9.4	0.49			
Status of extracranial disease			`Not applicable		
No progression or NED	26	*		*	*
Progression	5.1	<0.00001		2.157 [1.384-3.360] (p=0.001)	2.108 [1.358-3.274] (p=0.001)
New metastatic cancer diagnosis	11.4	*		*	*
Predicted median survival from GPAs		<0.00001	0.905 [0.866-0.946] (p<0.0001)	0.943 [0.897-0.991] (p=0.020)	0.920 [0.880-0.962] (p=0.001)
≤6.0 months	5.1				
>6-12 months	11				
>12 months	23.4				
Number of metastases		0.84	1.003 [0.980-1.027] (p=0.77)	Not included in the model	Not included in the model
5-10	7.8				
11-20	7.2				
>20	12.1				
Net GTV ¥ of metastases		0.32	1.007 [0.991-1.024] (p=0.40)	Not included in the model	Not included in the model
≤2 cc	9.4				
>2-5 cc	10				
>5-10 cc	5.4				
>10-20 cc	9				
>20 cc	3				

Twenty-seven patients died ≤2 months from SRS (n=21) or survived >2 months but opted against post-SRS follow-up imaging and further cancer therapy (n=6, with OS=2.1-5.8 months). Causes of deaths among these ‘poor survivors’ included extracranial (n=16) or intracranial (n=2) disease progression, infectious (n=2), renal failure (n=2), cardiac arrest (n=1), and unknown (n=4). ‘Poor survivors’, compared to others, were statistically more likely to have had: poorer KPS (p<0.001); progressive extracranial disease (p=0.012); and poorer GPA-predicted median OS (p=0.004). There were no appreciable differences in numbers or volumes of metastases between these two groups, nor in how many underwent systemic therapy prior to SRS (Table 1). Receipt of systemic therapy after SRS is confounded by OS.

Toxicity

Nine patients developed grade 2 (n=2) or grade 3-4 (n=7) radionecrosis/edema, of whom two underwent resection for radionecrosis 15 and 19 months after SRS. Radionecrosis vs hemorrhage (vs both) contributed to two deaths (grade 5 toxicity) 7-15 months after SRS.

New brain metastases

**Figure 3:**
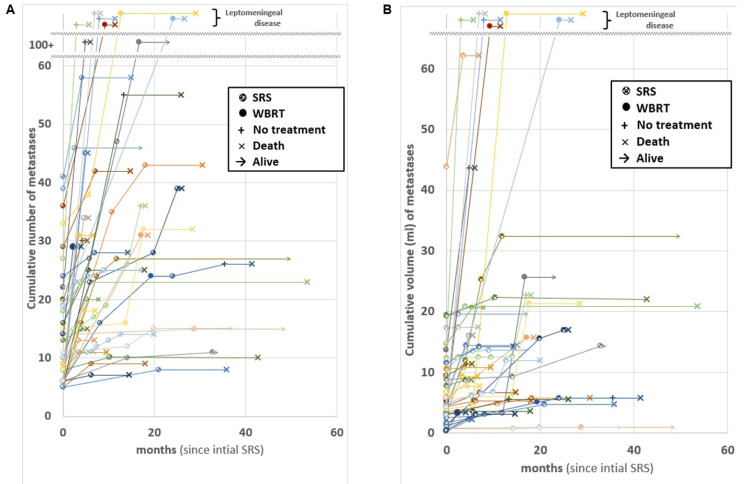
Cumulative number (A) and volume (B) of brain metastases versus time since the first SRS. Each individual plot represents a separate patient, with colored lines and markers to make it easier to follow the disease course. Brain metastasis velocity is reflected in the slopes of the lines. Therapies and endpoints (alive or dead) are denoted with specific symbols described in the legend. All patients underwent SRS at time zero. SRS: stereotactic radiosurgery; WBRT: whole-brain radiotherapy

Forty-two patients developed new brain metastases 2.3-28.7 (median 5.7) months after initial SRS, with 18 (43%) developing first/only new lesions beyond six months. Salvage therapies for new brain metastases included SRS (n=31 patients), WBRT (n=7, of whom four received WBRT as first salvage therapy), resection (n=1), and no additional therapy (n=7, of whom three had leptomeningeal disease). Seven patients developed leptomeningeal disease (five as first intracranial recurrence). Specifically for new metastases (i.e., not accounting for treatments of local recurrence), among 31 who received a second SRS, 13 received a third SRS (one who underwent resection prior to third SRS, and another who received WBRT for third treatment/second salvage), of whom five received a fourth SRS, of whom two received a fifth SRS. These numbers were too small to adequately analyze BMVs following first salvage therapy. Figure 3 depicts cumulative number (panel A) and volume (panel B) of brain metastases vs time since first SRS, with salvage therapies denoted by specific symbols. This figure is intended to be descriptive. BMV and vBMV plateau in some patients. Many with OS<12 months had high initial BMV (gauged by slopes of lines). However, some patients with initially high BMV or vBMV have relatively long OS. Melanoma vs other sites was not significantly associated with BMV (p=.13) or vBMV (p=.11).

Twenty-eight patients with minimum six-month imaging follow-up developed no new brain metastases (BMV=vBMV=0) at 8.2-74.7 (median 23.4) months. Thirty-seven patients developed new brain metastases (without leptomeningeal progression for 1st intracranial recurrence). Among these 37, BMV ranged from 0.4 to 183.6 (median 22.1) lesions/year and vBMV ranged from 0.01 to 73.5 (median 2.8) cc/year. There were no apparent trends with the initial brain metastasis number or net GTV with either BMV or vBMV, with linear and polynomial regressions being relatively flat and R-squared values <0.1.

BMV and vBMV, as univariable continuous or discrete variables, significantly correlated with OS (Table 3). On Cox regression including only BMV and vBMV, BMV remained significant (p=0.018) while vBMV did not (p=0.74). On multivariable Cox regression with BMV, vBMV, presence of progressive disease and GPA-predicted median OS, no variables were significant. In a separate model omitting vBMV, BMV was significant (p<0.0001) with a HR of 1.017 per lesion. vBMV was not significant on any multivariable Cox regression that included BMV (additional data not shown). Specifically for melanoma, BMV (p=0.050) and vBMV (p=0.046) were significant on univariable Cox regressions, whereas GPA-predicted median OS (p=0.13) and extracranial disease progression (p=0.94) were not.

**Table 3:** Survival outcomes and factors associated with survival among those evaluable for brain metastasis velocity BMV: brain metastasis velocity; CI: confidence interval; GPA: graded prognostic assessment; HR: hazard ratio; NED: no evidence of disease; OS: overall survival; vBMV: volume-based brain metastasis velocity * The survivals between these subgroups were not significantly different on the log-rank test (p=0.99). When these two groups merged (as was done in the study by Farris et al. [19]), the median survival was 35.9 months † Predicted median OS and progressive extracranial disease were each significant (p<0.02) in separate two-variable Cox regression analyses with BMV (data not shown), suggesting that they are somewhat interdependent prognostic factors. * analysis not done (comparison focused on progressive extracranial disease vs no progression at the time of brain metastases)

	Number of patients	Median OS	Univariable	Univariable Cox regression	Multivariable Cox regression (model 1)	Multivariable Cox regression (model 2)
		(months)	log-rank p value	HR [95% CI] (p value)	HR [95% CI] (p value)	HR [95% CI] (p value)
BMV:			0.003	1.022 [1.013-1.032] (p<0.0001)	1.017 [0.996-1.038] (p=0.11)	1.017 [1.008 -1.028] (p<0.0001)
0 per year *	28	26.5				
>0-<4 per year *	8	35.9				
4-13 er year	6	14.3				
>13 per year	23	15				
vBMV:			0.009	1.041 [1.019-1.063] (p<0.0001)	1.002 [0.955-1.053] (p=0.91)	Not included
0 cc per year	28	26.5				
>0-≤1 cc per year	10	28.5				
>1-5 cc per year	13	20				
>5 cc per year	14	7.8				
Predicted median OS from GPAs			0.011	0.916 [0.863-0.972] (p=0.004)	0.941 [0.884-1.003] (p=0.061)	0.942 [0.886-1.002] (p=0.058) †
≤6.0 months	27	14.3				
>6-12 months	7	28.5				
>12 months	31	29.1				
Status of extracranial disease				Not applicable		
NED (n=2) or No progression	16	29	*		*	*
Progression	14	9.4	0.0001		1.841 [0.816-4.153] (p=0.14)	1.850 [0.824 -4.154] (p=0.14) †
New metastatic cancer diagnosis	35	26	-*		*	*

Local control

Among 59 patients (presenting with 1,054 brain metastases), with ≥6 months of imaging follow-up, eight (with 176 initial brain metastases) developed one (n=5) or two (n=3) locally recurrent metastases. Thus, eight (13.5%) patients developed local recurrence of 11 (1%) brain metastases. Six patients received salvage SRS for local recurrence at 10-22 (median 18.7) months. Two died shortly after developing local recurrences at 8 and 15 months. Three patients, without local recurrence of lesions treated with initial SRS, developed local recurrences of lesions treated with subsequent SRS.

## Discussion

Our retrospective analysis of 116 patients, treated with SRS for five or more brain metastases, demonstrated that progressive extracranial disease at the time of brain metastases diagnosis and lower predicted median OS from GPAs were significantly adverse factors for OS. Among patients evaluable for new brain metastases, the number of metastases developing over time appeared to be more prognostic than a similar approach using volume.

While GPAs were not originally derived specifically from patients treated with SRS, they can reliably classify these patients into prognostic subgroups [31]. We opted to specifically analyze GPA-predicted median OS (as opposed to the GPA score), as GPA-predicted survivals vary across different cancer types. Notably, at the time of brain metastases diagnosis, all but two patients in our study had extracranial metastases, which all GPAs consider an adverse prognostic factor (zero points). Furthermore, all patients received zero GPA points for five or more brain metastases (for NSCLC, melanoma or RCC), four or more brain metastases (for GI cancers, or cancers without disease-specific GPAs) or more than one brain metastasis (for breast cancer). Consequently, no patients in this study fell into the highest GPA groups (scores of 3.5-4).

We generally offered SRS to patients with good KPS (or whose KPS was expected to improve) and with potential systemic options for extracranial disease. Nevertheless, there were (and are in general) patients who did not benefit from SRS and would have been better served with upfront supportive care alone [32]. Prognostic factors such as GPA-predicted median OS and status of extracranial metastases can inform shared (patient and physician) decision-making (i.e., SRS, supportive care, or reassessing after a short interval), conceding that these factors are probabilistic and not deterministic, and that our study lacks a non-SRS comparative arm. In a Polish study, early death after SRS was associated with: classic GPA score less than two; extensive extracranial metastases (more than three lesions with sum-diameters >3 cm); and serious neurological deficits (not specified) and/or requiring dexamethasone ≥4 mg daily. The authors recommended against SRS for patients with all three factors [33]. For our patients, we did not analyze the extent of extracranial disease, and neurologic symptoms were not significant for OS (attributable to most early deaths resulting from extracranial progression).

Patients presenting with five or more brain metastases have already demonstrated the propensity to develop multiple metastases. We postulated that vBMV would be more prognostic than BMV in this group, given that the volume of brain metastases appears to be more prognostic than the number [1,18]. However, our results did not demonstrate this. Possibly, BMV reflects underlying biologic aggressiveness and responsiveness to systemic therapy, while vBMV reflects growth rate. Larger studies, not restricted to patients presenting with five or more brain metastases, may better resolve this. To our knowledge, the only other published study of vBMV focused on patients treated with SRS from 2000-2013 for melanoma brain metastases, showing vBMV to be more prognostic than BMV for OS, new brain metastases, and need for salvage WBRT [28]. Our patient numbers were too small to separately analyze patients with melanoma.

The median OS in our report, subgrouped by BMV <4, 4-13 and >13 lesions/year, is numerically superior to the OS reported in other studies [19-21,34]. This may reflect more recent treatment (2018-2024) of our patients compared to those in studies by Farris et al. and McTyre et al. (2000-2013/2014) [19,20] and Yamamoto et al. (1998-2017) [21]. Our patients were treated in the era of high-resolution volumetric MRI and novel systemic agents with potential intracranial efficacy (i.e., immunotherapy, targeted therapies, and drug-antibody conjugates). Immunotherapy has been correlated with lower BMV and improved OS [35]. A series from U. Pittsburgh reported better OS than what we report here [36], likely reflecting differences in patient selection. Notably, 15% of their patients had undergone prior WBRT (which was not allowed in early studies on BMV, as WBRT was an endpoint) and all underwent multiple courses of SRS from 2013-2020, thus selecting patients living long enough to develop new metastases.

We separately analyzed patients with BMV=0. The BMV=0 group contains a mix of patients with low (to possibly no) propensity to develop new brain metastases, and those with no opportunity to develop new brain metastases prior to death. The Wake Forest group showed that, for patients alive more than two years after SRS, BMV=0 was significantly associated with single brain metastases and Caucasian race; OS was not specifically analyzed [37]. In our analysis, BMV=0 patients did not experience significantly different OS than patients with a 0<BMV<4 (Table 3), which (to our knowledge) is a new finding. In the aforementioned US studies [19,20], the BMV <4 group included BMV=0 patients, while in the Japanese study [21], all patients underwent SRS for new lesions and therefore all had BMV>0. As in the US studies on BMV [19,20], we did not restrict BMV calculations to those who underwent a second SRS (i.e., we opted to include patients who underwent salvage WBRT or no salvage therapy).

Factors associated with increased BMV from the US pooled analysis included melanoma histology and number of initial brain metastases [20]. Farris et al. [19] reported that two or more brain metastases at presentation, number of metastases (continuous variable) and melanoma were associated with higher BMVs, while Her2+ breast cancer was associated with lower BMVs. Melanoma was not significantly associated with a higher BMV in our patients. In the series by Yamamoto et al., small cell lung histology (which was excluded from our cohort) was associated with higher BMV. In a study of patients with five to 15 brain metastases, BMV was similar among those presenting with five to nine vs ten to 15 lesions [38]. In another study of patients with one to 15 brain metastases, BMV was significantly (p<0.01) lower among patients with one vs two to four vs five to 15 brain metastases [39]. In our series of patients presenting with five or more brain metastases, there was no discernible relationship between initial number or volume of brain metastases and BMV or vBMV. BMV acceleration or deceleration over multiple time points could also be prognostic [19], though our patient numbers were too small to evaluate this. Nevertheless, it is evident that BMV and vBMV plateau in some long-term survivors as reported by the Wake Forest group [37].

Two large studies examined the velocity of brain metastases from time of initial cancer diagnosis to initial brain metastases- termed iBMV [40,41]. We opted against analyzing iBMV since many patients in our cohort presented with newly diagnosed cancer synchronously with brain metastases.

Limitations of our study include relatively small numbers of patients (compared to larger institutions and pooled databases), a single-institution cohort (with potential selection biases), subjectivity in assigning KPS [31], heterogeneous cohorts, and retrospective design (without standardized upfront or salvage therapies). Because we selected patients receiving upfront SRS, we could not assess outcomes following upfront WBRT or deferral of radiotherapy in favor of supportive care or CNS-active systemic therapy (a treatment paradigm actively being investigated [42-44]). Some subgroup analyses of BMV/vBMV were not feasible due lack of statistical power. Strengths include the large (for a single-institution analysis) sample size and modern-era (2018-2024) cohort of patients treated with contemporary systemic therapies.

## Conclusions

We demonstrated that clinical factors can inform treatment decision-making for patients with multiple brain metastases. In this descriptive, hypothesis-generating analysis, progressive extracranial disease at the time of brain metastasis diagnosis and lower predicted median OS based on GPA scores were significantly associated with worse outcomes, including survival of less than two months.

Future studies with larger, multi-institutional cohorts are needed to refine criteria that guide treatment selection for newly diagnosed brain metastases. Such efforts should include patients receiving a range of initial treatment approaches, rather than focusing solely on those treated with SRS. Moreover, incorporating radiomic and genomic data, along with artificial intelligence-based predictive modeling, may further enhance prognostic accuracy and deepen our understanding of outcome determinants.
